# Leukaemic alterations of *IKZF1* prime stemness and malignancy programs in human lymphocytes

**DOI:** 10.1038/s41419-018-0600-3

**Published:** 2018-05-09

**Authors:** Zhen Li, Shui-Ping Li, Ruo-Yan Li, Hua Zhu, Xia Liu, Xiao-Lin Guo, Li-Li Mu, Jie-Jing Cai, Fan Bai, Guo-Qiang Chen, Deng-Li Hong

**Affiliations:** 1 Key Laboratory of Cell Differentiation and Apoptosis of Ministry of Education, Department of Pathophysiology and Ruijin Hospital, Shanghai Jiao Tong University School of Medicine (SJTU-SM), Shanghai, 200025 China; 2 Biodynamic Optical Imaging Center (BIOPIC), School of Life Sciences, Peking University, Beijing, China; 3 Key Laboratory of Pediatric Hematology and Oncology Ministry of Health, Department of Hematology/Oncology, Shanghai Children’s Medical Center, SJTU-SM, Shanghai, China; 4 Department of gynaecology and obstetrics, Huangshi Aikang Hospital of Hubei Province, Huangshi, China; 5 Shanghai Key Laboratory of Reproductive Medicine, SJTU-SM, Shanghai, China

## Abstract

Somatic cells acquire stem cell-like properties during cancerous transformation; however, mechanisms through which committed cells develop stemness and malignancy remain largely unknown. Here we uncovered upregulated stem cell program in leukaemic lymphoblasts of patients with *IKZF1* alterations by analysing the archived gene-expression profiling datasets. We then used a frequent *IKZF1* deletion, IK6, as a model via transduction into human primitive haematopoietic cells, followed by xenotransplantation in mice. Immunophenotypically defined stem, pro-B, and immature/mature (IM/M)-B cells were collected from primary recipients for functional assay and transcriptome profiling. Successful reconstitution in secondary recipient mice revealed the stemness of IK6^+^ pro-B and IM/M-B cells. Upregulated stemness and malignancy programs in IK6^+^ cells confirmed IK6 effects. Interestingly, these programs corresponded to distinct canonical pathways. Remarkably, the pathway profile mapped in the modelled cells well mirrored that in patients’ leukaemic cells; therefore, our study provides a seminal insight into the cancerous reprogramming of somatic cells.

## Introduction

Compelling evidence has demonstrated that malignant somatic cells over a wide range of immune phenotypes can propagate cancer by acquiring stem cell properties^1–6^. Understanding how these somatic cells are reprogrammed to gain stemness and malignancy is important for cancer pathogenesis as well as cell reprogramming to avoid malignancy. In the present study, by assessing the biological effect of leukaemic alterations of *IKZF1* in committed lymphocytes in humans, we provide a substantial insight into the functional and molecular bases of the mechanism by which somatic cells are reprogrammed to become cancerous.

*IKZF1* encodes a transcription factor Ikaros, which is a zinc finger DNA-binding protein. *IKZF1* is widely expressed throughout the haematopoietic system^7–9^, and it is functionally involved in the early lymphoid development and in governing the developmental pathway of lymphoid or myeloid lineage from multipotent progenitors^10–13^. *IKZF1* alterations are recurrent in acute lymphoblastic leukaemia (ALL) and chronic myeloid leukaemia that progress into lymphoid blast crisis^14–19^. Among these alterations, a frequent deletion involving exons 3–6 (e3–e6) of *IKZF1* results in the expression of an isoform IK6. IK6 lacks the DNA-binding domain in the N-terminal and retains the dimerisation domain in the C-terminal. Previous studies have shown that IK6-expressing cells develop a stem cell-like property that was mainly characterised using colony-forming assays in vitro^20–22^; however, it remains unclear whether *IKZF1* alterations confer stemness and/or malignancy in committed B cells, as functionally characterised in leukaemic lymphoblasts within a wide range of immunophenotypes^1–4^, and how the underlying transcriptional programs are primed.

In this study, we analysed the archived gene expression profiling datasets of patients with leukaemia and uncovered the stem cell program that was activated in leukaemic cells with *IKZF1* alterations. We then convincingly assessed the functional role of leukaemic IK6 as a single event in human committed lymphocytes using an advanced xenotransplantation model^4, 23, 24^. We also systemically analysed the programs in the whole transcriptome activated by IK6 expression. We confirmed the self-renewal potential of IK6 expressing lymphocytes in vivo. We demonstrated the identical programs of stemness and malignancy as well as the corresponding signalling pathways activated in IK6-expressing lymphocytes that were traced down to the transcriptomes of patients’ leukaemic cells; therefore, our study sheds new light on the mechanism underlying the reprogramming of somatic cells into cancerous cells.

## Results

### Stem cell programs uncovered in leukaemic lymphoblasts with *IKZF1* alterations

**Fig. 1:**
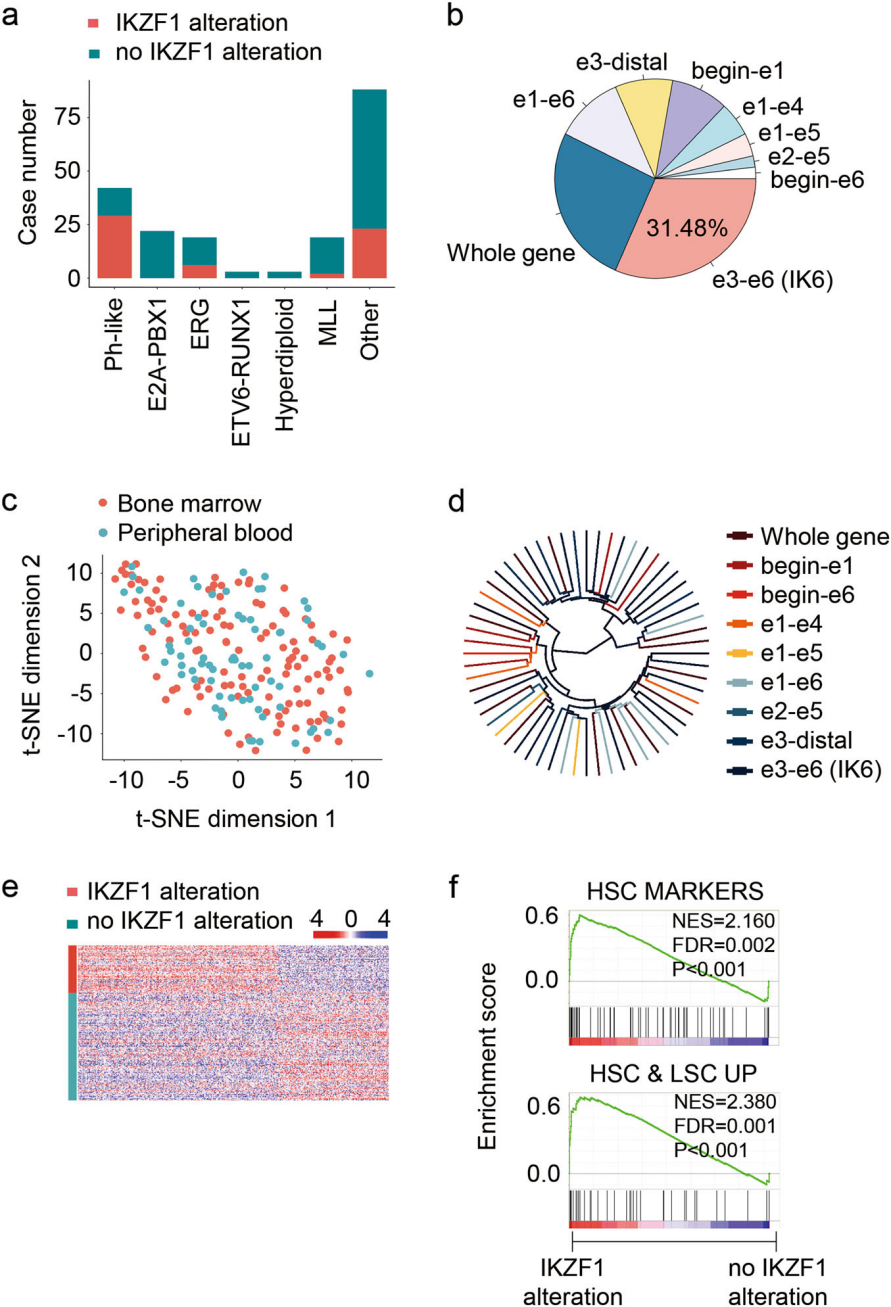
Stem cell programs are upregulated in leukaemic cells with *IKZF1* alterations. **a** Occurrence of *IKZF1* alterations in subtypes of acute lymphoblastic leukaemia (ALL) in patients covered by the GSE11877 dataset^25^. **b** Classification of *IKZF1* alterations in patients of the GSE11877 dataset, according to the ranges of altered exons. **c** *t*-Distributed Stochastic Neighbour Embedding analysis of the data of samples covered by the GSE11877 dataset and mapped sample sources (peripheral blood and bone marrow). *Note*: no differences between the two sources were detected. **d** Unsupervised hierarchical clustering analysis of the expression profiles in the samples with IKZF1 mutants. *Note*: no exclusive clusters were observed. **e** Heat map of 368 differentially expressed genes (*p* < 0.05) between leukaemic cells of patients with and without *IKZF1* alterations. **f** Gene Set Enrichment Analysis (GSEA) of the GSE11877 dataset contrasting samples with and without *IKZF1* alteration. *Note*: haematopoietic stem cell (HSC) and leukaemic stem cell (LSC) programs were upregulated in cells with *IKZF1* alterations

The detailed clinical and omics data of patients with leukaemia collected in the Therapeutically Applicable Research to Generate Effective Treatments (TARGET) program and Pediatric Cancer Genome Project (PCGP) provide excellent resources for exploring the mechanisms involved in somatic cell alterations^18, 19, 25^. A metadata summary of 1781 patients from the consortium revealed a high recurrence of *IKZF1* alterations in all subgroups of patients with leukaemia (sFig. 1a). Among these patients, the archived whole transcriptome profiling dataset GSE11877 covered 196 patients within several subgroups and 60 patients with *IKZF1* alterations (Fig. 1a,b; sTable 1). Considering that these samples were from patients’ bone marrow and/or peripheral blood, dimension reduction with the t-Distributed Stochastic Neighbour Embedding of the dataset resulted in a uniformly distributed sample; no cluster tendency was detected when the annotated tissue sources were mapped among total 196 samples of the dataset (Fig. 1c) or the 60 samples with *IKZF1* alterations (sFig. 1b). Unsupervised hierarchical clustering analysis of the expression profiles in the samples with *IKZF1* mutants was then performed. No exclusive clusters were observed indicating no significant difference between *IKZF1* mutations (Fig. 1d). Thus, differential gene expression analysis was conducted for patients with or without *IKZF1* alterations, showing consistent differences between them. Significantly, 368 genes were found to be differentially expressed (Fig. 1e). Gene set enrichment analysis (GSEA) was then performed on this difference, which revealed that haematopoietic and leukaemic stem cell (HSC and LSC, respectively) programs were prominently enriched in patients with *IKZF1* alterations^26, 27^ (Fig. 1f). Similar results were obtained within the whole transcriptome profiling dataset generated via the RNA sequencing (RNA-seq) samples obtained from patients with leukaemia from a PCGP cohort^26, 27^ (sFig. 1c, sTable 2).

Together, these results suggest that *IKZF1* alterations, as a single event, could prime stem cell programs in lymphoblasts for oncogenesis.

### IK6 expression confers stemness in human committed lymphocytes

**Fig. 2:**
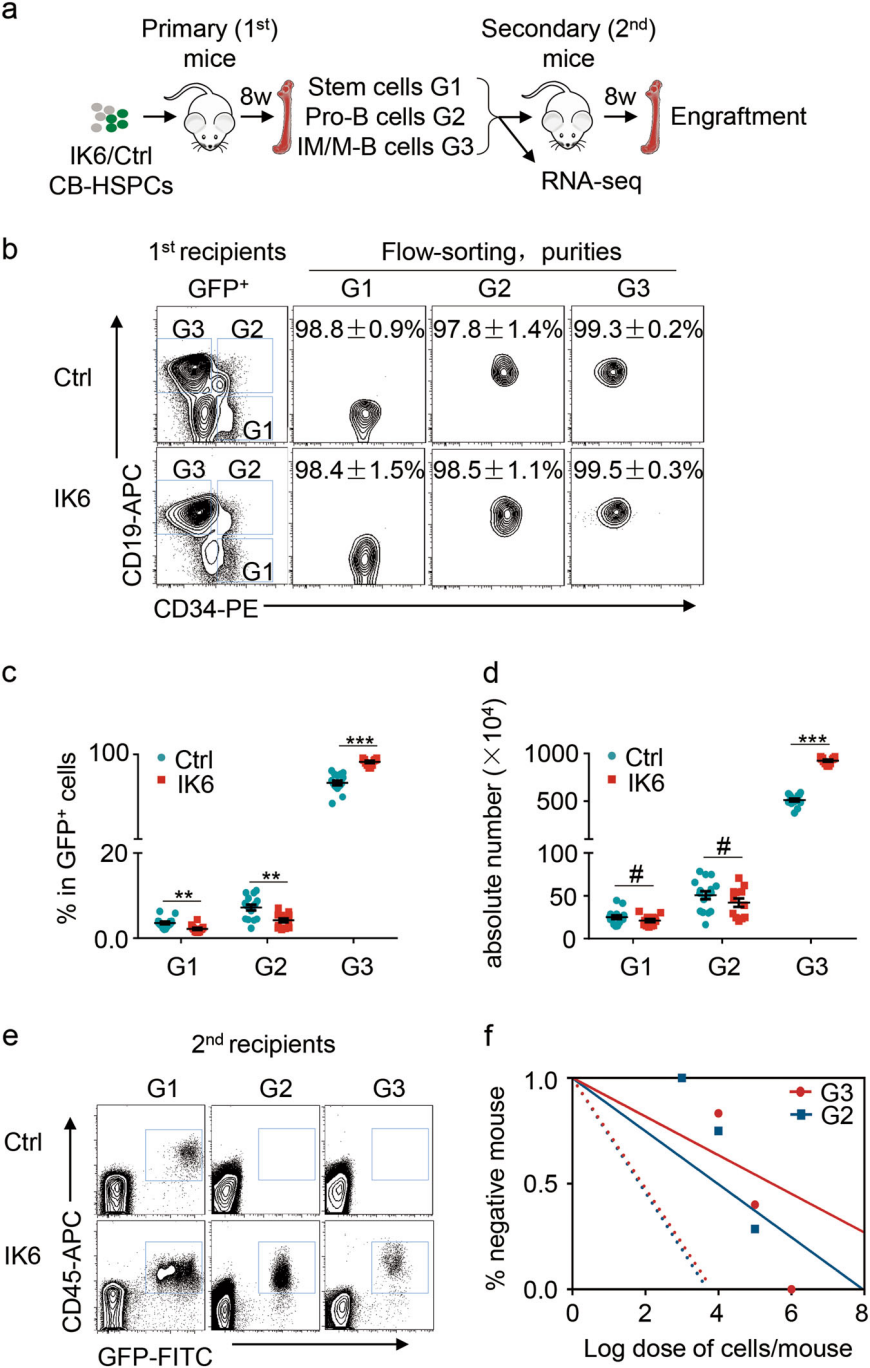
IK6 expression confers stemness in human committed lymphocytes. **a** Overall experimental design. **b** Flow cytometric analysis of B cell development of the engrafted GFP^+^ cells in primary recipient mice at 8 weeks after transplantation. Stem cells enriched CD34^+^CD19^−^ (G1) fraction, Pro-B in CD34^+^CD19^+^ (G2) fraction and immature/mature (IM/M)-B in CD34^−^CD19^+^ (G3) fraction are shown. These fractions were flow sorted and purities were detected. **c**, **d** Percentages (**c**) and absolute cell numbers (**d**) of G1, G2 and G3 engrafted in Ctrl and IK6 mice. All data are presented as the mean ± SD. ^#^*P* > 0.05, ^*^P < 0.05, ^**^P < 0.005, ^***^P < 0.001. **e** Flow cytometric analysis of the reconstitution (engraftment) of G1, G2 and G3 of Ctrl or IK6 cells in secondary recipient mice. The representative plots are shown. **f** Severe combined immunodeficiency (SCID)-repopulating cell (SRC) assay by limiting dilution; the estimated SRC frequencies (solid lines) for G2 and G3 were 1/6.6 × 10^4^ and 1/1.0 × 10^5^, respectively. Dotted lines indicate 95% confidence intervals

The complete coding sequence of IK6 was amplified by polymerase chain reaction (PCR) from an ALL cell line Sup-B15 (sFig. 2a) and cloned into lentiviral expression vector pHR-SIN-CSIGW to investigate the biological effect of leukaemic alterations of *IKZF1* and the underlying programs in HSCs (sFig. 2b). IK6 expression was confirmed in human umbilical cord blood (CB) CD34^+^ cells by Western blotting without effects on the expression of wild-type *IKZF1* (sFig. 2c). The lentivirus of IK6 and the control vector (Ctrl) were respectively used to infect human CB haematopoietic stem and progenitor cells (CB-HSPCs, Lin^−^CD34^+^CD38^−^) followed by xenotransplantation into an advanced immunodeficient mouse model^4, 23, 24^. Reconstituted cell fractions in the primary recipients (1st) containing stem cells (G1), pro-B cells (G2) or immature and mature (IM/M)-B cells (G3) were collected for functional assessment in secondary recipients (2nd) and whole transcriptomic analysis by RNA-seq (Fig. 2a).

The engraftment in 1st mice was analysed using flow cytometry at 8 weeks after transplantation on the basis of the expression of human CD45, which revealed higher engraftment in IK6 mice than in Ctrl mice (sFig. 2d). Infection efficiencies (the percentage of GFP^+^ cells) in the input and engrafted cells in 1st mice were also analysed by flow cytometry (sFig. 2e, f), which are presented in sFig. 2f. In the engrafted GFP^+^ cells, the lineage distribution of B cells (CD19^+^), T cells (CD3^+^) and myeloid cells (CD33^+^) was analysed. The results indicated that B cells were increased, whereas T cells and myeloid cells were decreased in IK6 mice compared with those in Ctrl mice (sFig. 2g), which is consistent with the clinical findings and the results reported in other models^14, 20, 22, 25^.

B cell development in engrafted GFP^+^ cells was further analysed by the combined use of cell surface markers CD19 and CD34 (Fig. 2b). On the basis of the archived developmental profiles of human HSC and progenitor cells in xenograft models^28–30^, stem cells should be enriched in CD34^+^CD19^−^ (G1) fraction, pro-B cells in CD34^+^CD19^+^ (G2) fraction and IM/M-B cells in CD34^−^CD19^+^ (G3) fraction. G1 and G2 in IK6 mice were statistically lower than those in Ctrl mice, whereas G3 was markedly higher in IK6 mice (Fig. 2c). When the absolute cell numbers of these groups were calculated, G1 and G2 appeared to be not significantly different between IK6 and Ctrl mice, whereas G3 in IK6 mice was markedly increased, indicating that IK6 expression led to a great expansion in G3 group (Fig. 2d).

G1, G2 and G3 fractions in IK6 and Ctrl cells were flow sorted with high purities (Fig. 2b) and used in parallel for transplantation into 2nd mice to assess their reconstitution ability (standing for stemness) and for RNA-seq to analyse the underlying transcriptome (Fig. 2a). Serial numbers of G1 (10^2^, 10^3^, 10^4^), G2 (10^3^, 10^4^, 10^5^) and G3 (10^4^, 10^5^, 10^6^) cells were transplanted into 2nd mice, respectively. At 8 weeks after transplantation, bone marrow (BM) cells were harvested to analyse the repopulating potential of each group. As expected, G1 cells of both IK6 and Ctrl could reconstitute in 2nd mice containing stem cells. Surprisingly, IK6 G2 and G3 cells carrying committed B cell markers could repopulate well in 2nd mice, whereas the immunophenotypically corresponding Ctrl G2 and G3 cells could not repopulate, which was consistent with previous reports^24, 31^ (Fig. 2e, Table 1). Frequencies of the SCID-repopulating cell (SRC) in IK6 G2 and G3 were calculated using the L-Calc^TM^ software, which showed no significant difference between the two groups (Fig. 2f, Table 1).

**Table 1:** Secondary transplantation of G2 and G3 of Ctrl or IK6 cells

	Group	Cell dose	Engrafted mice/transplanted mice	SRC frequency
				1 SRC per *n* cells
				(95% CI)
Ctrl	G3	1 × 10^6^	0/6	–
		1 × 10^5^	0/22	
		1 × 10^4^	0/10	
	G2	1 × 10^5^	0/8	–
		1 × 10^4^	0/8	
		1 × 10^3^	0/6	
IK6	G3	1 × 10^6^	4/4	1.0 × 10^5^
		1 × 10^5^	12/20	(5.8 × 10^4^~1.7 × 10^5^)
		1 × 10^4^	2/12	
	G2	1 × 10^5^	5/7	6.6 × 10^4^
		1 × 10^4^	2/8	(2.9 × 10^4^~1.5 × 10^5^)
		1 × 10^3^	0/6	

These convincing results of functional analyses indicated that IK6 expression indeed conferred stemness in committed B lymphocytes with variant immunophenotypes, as observed in cells of patients with ALL^2, 3^.

### IK6 expression primed several leukaemic programs in lymphocytes

To investigate programs activated by IK6 expression in lymphocytes, the transcriptomes were comparatively analysed using RNA-seq among G1, G2 and G3 of IK6 and Ctrl mice isolated from 1st recipients’ BM (Fig. 2a,b) and nontransplanted B cells (CD19^+^) as well as HSCs (Lin^−^CD34^+^CD38^−^) isolated from human umbilical cord blood (CB B cells, CB HSCs).

**Fig. 3:**
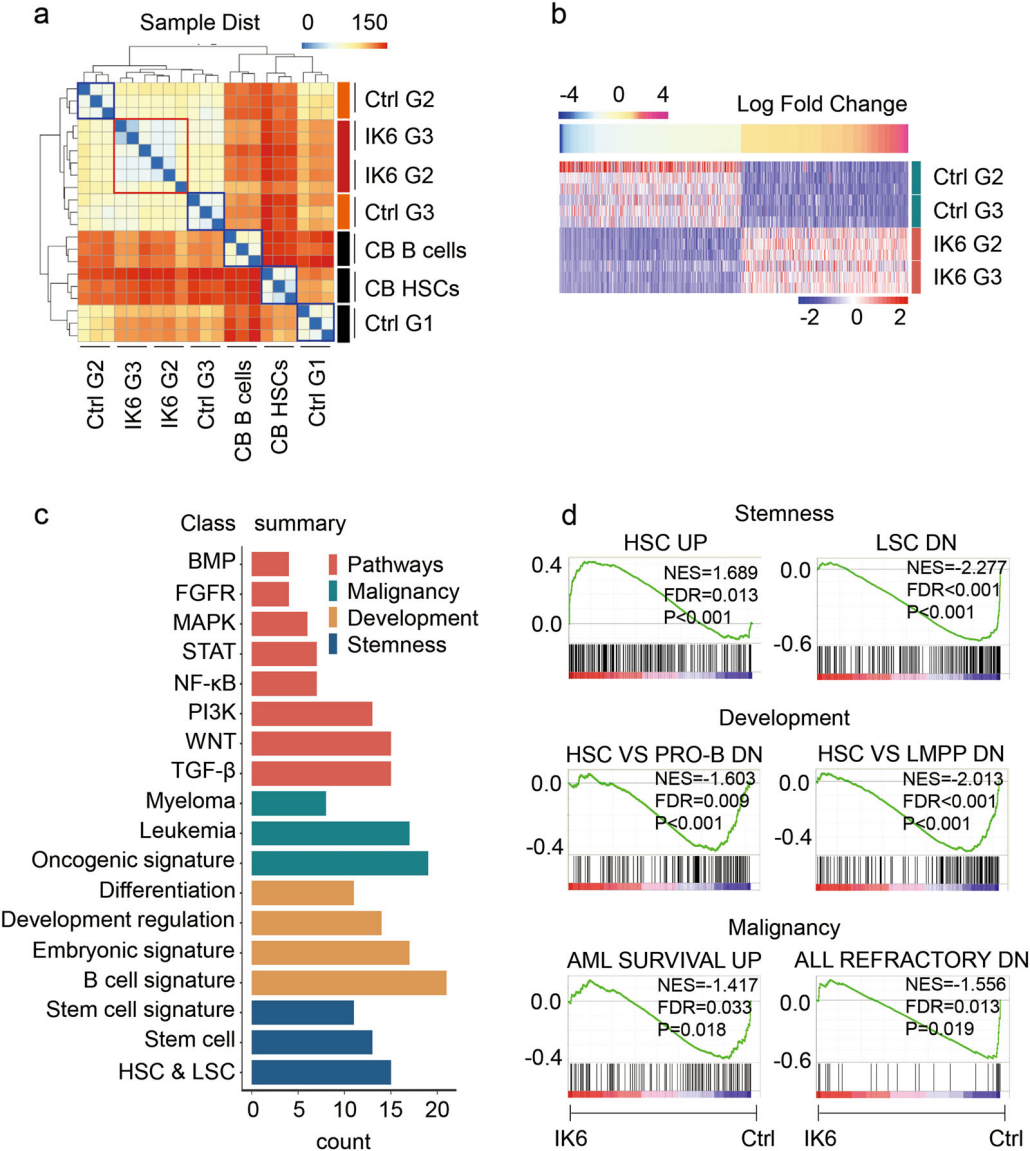
IK6 expression primes several leukaemic programs in human lymphocytes. **a** Unsupervised clustering analysis of the whole transcriptome between the labelled samples. Note that IK6 G2 and G3 tended to be close (in the red square). **b** Heat map showing differentially expressed genes among IK6 groups (IK6 G2 plus IK6 G3) and control groups (Ctrl G2 plus Ctrl G3). The genes that changed twofold or more are shown. **c** GSEA between IK6 groups (IK6 G2 plus IK6 G3) and control groups (Ctrl G2 plus Ctrl G3). Note that the enriched gene sets could be classified into stemness, development, malignancy and signalling pathways, outlining the possible roles of IK6. **d** The typical enrichment plots of GSEA results of (**c**) stemness, development and malignancy

Consistency among the three samples in independent experiments within each group indicated that the high quality of RNA-seq data was sufficient for further analyses (sFig. 3a). A heat map of differentially expressed genes indicated three important aspects of the bioinformation (Fig. 3a). First, well clustering of the three samples at intragroup level further proved the reliability of the data (blue and red squares). Second, IK6 G2 and G3 tended to be close to each other (red square), which reflected the functional phenotype of the similar property of stemness (Fig. 2f). Third, IK6 and Ctrl showed a short distance but convincingly distinct phenotype, indicating the significance of further intergroup analysis of differential gene expression.

We next analysed the Ctrl groups (G1, G2 and G3) in comparison with the groups flow sorted from CB cells (i.e., CB HSCs and CB B cells) to assess whether the Ctrl groups could recapitulate the B-lineage development stages in our xenograft model. Indeed, an unsupervised hierarchical clustering analysis of the whole transcriptome revealed that Ctrl G1 was close to CB HSCs and that Ctrl G2 and Ctrl G3 were close to CB B cells (sFig. 3b). To further confirm these results, GSEA of the whole transcriptome among Ctrl G1, G2 and G3 was conducted. Stem cell program was negatively enriched, whereas B lymphocyte progenitor program was positively enriched in Ctrl G2 compared with those in Ctrl G1; these results were statistically significant (sFig. 3c). B lymphocyte programs were significantly enriched in Ctrl G3 compared with those in Ctrl G2 (sFig. 3d). Together, these results confirmed that our xenograft model of the recipient’s BM environment was suitable for HSC maintenance and B-lineage development, as reflected by the immunophenotypes. Thus, these data provide a suitable basis to analyse the programs mediated by IK6 expression in the corresponding groups.

We then assessed whether IK6 expression conferred dominant-negative (DN) effects on *IKZF1* in our models. The top 100 genes that showed possible physical interaction with *IKZF1* were retrieved from GeneMANIA. Unsupervised hierarchical clustering analysis of these genes in our RNA-seq dataset revealed minimal differences between IK6 and Ctrl samples (sFig. 3e). Genes that are potentially regulated by *IKZF1* were then retrieved from ENCODE_TF_ChIP-seq and analysed in our dataset. In total, 239 of these genes showed significant differential expression between the IK6 and Ctrl samples (sFig. 3f). Together, these results indicate that IK6 exert DN effects on *IKZF1*, which are consistent with the results of other studies^21, 22^.

We then compared transcriptomes among IK6 groups (G2 and G3) and Ctrl groups (G2 and G3) because the heat map revealed that IK6 expression assimilates the transcriptomes of G2 and G3 (Fig. 3a, red square). IK6 expression might induce similar changes in G2 and G3 that mediate programs underlying its biological effect. Indeed, differential gene expression analysis revealed the existence of common altered genes among the IK6 groups (G2 and G3) and Ctrl groups (G2 and G3). Genes altered twofold or more are shown in Figure 3b.

We then conducted GSEA of the IK6 groups (G2 and G3 together) and Ctrl groups (G2 and G3 together). Intriguingly, differentially expressed genes were enriched in the gene sets (also called ‘programs’) that have been archived in association with leukaemogenesis and hallmarks of cancer cells, and these gene sets could be classified into stemness, development (including differentiation), malignancy and canonical signalling pathways, which outlined the possible roles of IK6 expression in lymphocytes during oncogenesis (Figure 3c). The typical enrichment plots are shown in Fig. 3d.

### IK6 expression activates distinct pathways to reprogramme lymphocytes

**Fig. 4:**
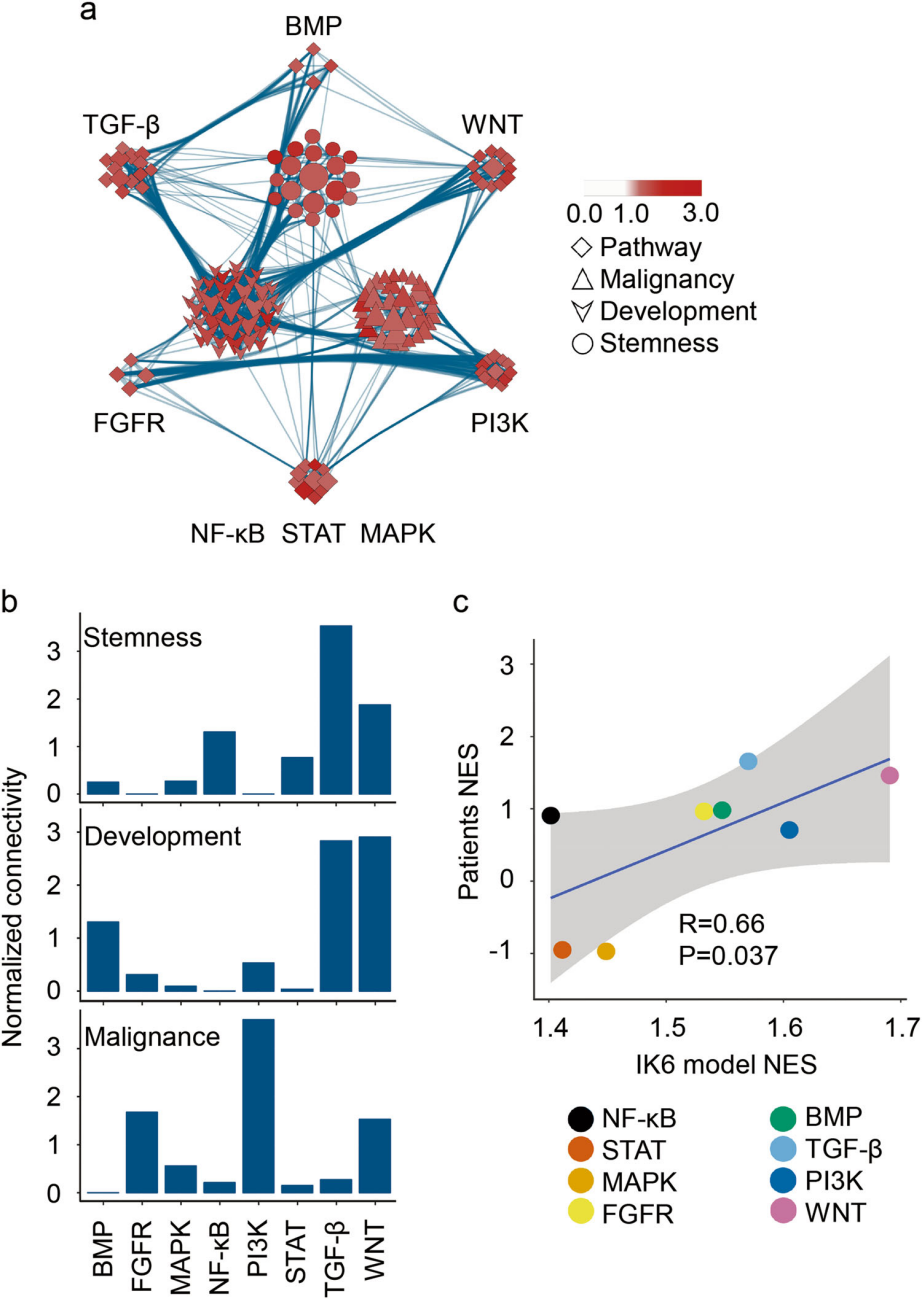
IK6 expression activates distinct pathways in reprogramming lymphocytes. **a** The network analysis of the GSEA results between the biological effects and signalling pathways activated by IK6 expression. **b** Qualification of the association between the biological effects and signalling pathways based on the network data of (**a**). **c** Correlation analysis of the activated canonical pathways between the model and patient GSE11877 cohort. Significant correlation was shown with a correlation coefficient of 0.67 and a *P*-value of 0.037

To dissect the correspondence between the enriched pathways and the three gene set groups as stemness, development and malignancy (Fig. 3c), we generated a network from the GSEA results to visualise the association among the enriched gene set groups (Fig. 4a, see also details in ‘Methods’). By viewing the network, we found that stemness and development (including differentiation) were tightly coupled and that both were loosely linked with malignancy (Fig. 4a). Further observation revealed that these gene set groups were disproportionately associated with the enriched pathways. Accordingly, the pathways could be classified into two clusters as follows: cluster 1, which was closely associated with stemness and differentiation, including TGF-β, WNT and BMP pathways and cluster 2, which was closely associated with malignancy, including PI3K, FGF, NF-κB, STAT and MAPK (Fig. 4a) pathways. By quantifying these associations, we uncovered that stemness was most closely associated with TGF-β signalling pathway, development was most closely associated with TGF-β and WNT signalling pathways and malignancy was most closely associated with the PI3K signalling pathway (Fig. 4b).

To further investigate whether the pathway profile mapped in our modelled cells mirror the reality in the patient ALL cells, we compared the enrichment of conserved pathways between the model and GSE11877 cohort patients. Indeed, a similar profile was identified, with a statistical correlation coefficient of 0.66 and a *P*-value of 0.037 (Fig. 4c).

Together, the findings of the present study posit a mechanism underlying the reprogramming of somatic cells into cancerous cells via *IKZF1* alterations (including IK6) that might affect distinct pathways to alter corresponding programs and eventually confer stemness and malignancy in the target cells that could be a committed non-stem cell.

## Discussion

In the present study, an identical stem cell program that was commonly activated in the leukaemic lymphoblasts of several subgroups of ALL patients with *IKZF1* alterations was revealed. Convincing results from our functional assays in vivo demonstrated that IK6 expression (a frequent *IKZF1* alteration) could confer the stem cell-like property (i.e., self-renewal ability) to committed lymphocytes. Interestingly, the systemic analysis of the programs in the whole transcriptome activated by IK6 expression revealed that *IKZF1* alterations, as a single event, could prime programs of stemness, impaired differentiation and malignancy in the lymphocytes within a wide range of immunophenotypes. Further analysis revealed that these activated programs were associated with the activation of distinct pathways. Stemness and differentiation were coupled with the TGF-beta and/or WNT pathways, and malignancy was coupled with the PI3K pathway. Together, these results indicate a model of oncogenesis wherein somatic cells are reprogrammed into cancerous cells via a single-drive mutation that could prime the target cells with several programs by synchronously activating distinct pathways and suggest that multiple features of cancer cells could be unnecessarily primed by distinct mutations.

Malignancy remains an undefined concept. Our gene set mapping network (Fig. 4a) implies that malignancy might indeed be a type of inflammation because the involved regulatory pathways of inflammation (i.e., PI3K, NF-κB, etc.) were more closely associated with malignancy.

In conclusion, the present study provides a paradigm to shed light on cancerous reprogramming in somatic cells; therefore, the proposed model might have great implications in investigating cancer pathogenesis as well as inducing dedifferentiation or transdifferentiating of somatic cells to avoid malignant formation.

## Materials and methods

### Patient data on *IKZF1* alterations

The archived gene expression profiling datasets on *IKZF1* alterations in patients with leukaemia used in this study were obtained from TARGET and the St. Jude Children’s Research Hospital, Washington University PCGP. The data from TARGET can be accessed at https://ocg.cancer.gov/programs/target or through the National Center for Biotechnology Information under accession number GSE11877. The data from PCGP can be accessed at the European Genome-phenome Archive under accession number EGAD00001001016. The *IKZF1* alteration status of the patients was collected from publications arising from TARGET or PCGP that described the results of the analyses of the patient data^18, 19, 25^. Differential gene expression analysis and GSEA were conducted with data on patients with explicit information of *IKZF1* alteration; the number of qualified samples (196 and 107 in GSE11877 and the EGAD00001001016 cohort, respectively) and the primary information on these patients are summarised in Supplementary Tables 1 and 2.

### Human umbilical CB cells

Human CB cells from anonymised healthy donors were purchased from the Cord Blood Bank in accordance with local ethical procedures. Total mononuclear cells were freshly isolated by Ficoll (GE Healthcare, UK) gradient centrifugation as previously described^4^. CD34^+^ fractions were separated using magnetic beads (STEMCELL Technologies, Vancouver, BC). HSPCs were flow sorted in the phenotype Lin^−^CD34^+^CD38^−^.

### Cloning of IK6 cDNA

Human IK6 cDNA, obtained by PCR from an ALL cell line Sup-B15, was cloned into a pHR-SIN-CSIGW lentiviral vector, which carries an emerald-GFP reporter, and was sequence verified^32^.

### Preparation of lentivirus and CB cell transduction

Lentivirus was produced and concentrated as previously described^4^. A high-titre virus of IK6-GFP and Ctrl-GFP was prepared by ultracentrifugation. Lin^−^CD34^+^CD38^−^ HSPCs were infected with lentivirus at a multiplicity of infection of 100 for 24 h in serum-free Stem/Span (Stem Cell Technologies, Canada) medium supplemented with growth factors (hSCF 50 ng/ml, hFlt3-L 50 ng/ml, hIL-6 10 ng/ml and hTPO 10 ng/ml; Peprotech, USA).

### Mice and transplantations

Nonobese diabetic/severe combined immunodeficiency (NOD/SCID) mice were purchased from Shanghai Laboratory Animal Company and maintained under sterile conditions at the animal facility in accordance with local regulations. Transplantations (2 × 10^4^ HSPCs/mouse) were performed by intratibial injections in mice aged 8–10 weeks after total-body irradiation with the sublethal irradiation of 150 cGy from a Cs137 source and treatment with 200 μg CD122 antibody through intraperitoneal injection^4, 23, 33^. The 1st mice were sacrificed at 8 weeks after transplantation. BM cells of the femurs, tibias and pelvises were collected, and the engrafted cells in the stem cell-enriched fraction (CD34^+^CD19^−^, G1), pro-B cells fraction (CD34^+^CD19^+^, G2) and IM/M-B cells fraction (CD34^−^CD19^+^, G3) were flow sorted for RNA-seq and transplantation in 2nd mice, respectively. The secondary transplantation was performed by limiting dilution in cell numbers. The engraftment was detected at 8 weeks after transplantation, and the SRC frequency was calculated using L-Calc^TM^ (STEMCELL Technologies, Vancouver, BC). All animal experiments were approved by the Experimental Animal Ethical Committee at Shanghai Jiao Tong University School of Medicine, China, and performed in accordance with the National Centre for the Replacement, Refinement & Reduction of Animals in Research, Animal Research: Reporting of in vivo experiments guidelines.

### Immunophenotypic analysis and flow sorting

BM cells from recipient mice were flushed with Iscove’s modified Dulbecco’s medium (IMDM, Gibco, USA) supplemented with 1% bovine serum albumin (BSA, Sigma-Aldrich, St. Louis, MO, USA). Cells were then treated with ammonium–chloride–potassium (ACK) solution (150 mM NH_4_CL, 1 mM KHCO_3_ and 0.1 mM EDTA; Sigma, Germany) at room temperature for 10 min to lyse red blood cells. After washing and resuspending in phosphate-buffered saline (PBS) + 0.5% BSA, cells were stained with the following antibodies at 4 °C for 30 min in the dark: CD45-APC or CD45-PE-Cy7 for engraftment; CD34-PE and CD19-APC for B cell differentiation and CD19-PE (B cells), CD3-PE (T cells) and CD33-PE (myeloid cells) for lineage distribution. All antibodies were obtained from BD Bioscience. Stained cells were analysed or sorted using flow cytometer (Aria II; BD, NJ, USA).

### RNA-seq

Total RNA was respectively extracted from CB HSCs, CB B cells, Ctrl G1, Ctrl G2, Ctrl G3, IK6 G2 and IK6 G3 groups (each with three biological replicates) using an RNeasy Mini Kit (Qiagen, Germany) according to the manufacturer’s protocol. Quality checks of the RNA product were performed using Fragment Analyzer^TM^ platform (Advanced Analytical Technologies, Inc.). RNA products of high quality (RNA quality number >7.0) were further used for library construction using the Illumina TruSeq RNA Prep Kit. Libraries were further quality checked and sequenced with the Illumina HiSeq 4000 platform, generating 2 × 150 bp paired-end (PE) reads.

### RNA-seq data processing

PE-cleaned reads were aligned to human reference genome hg19 (UCSC) using TopHat (v 2.1.1) with default parameters. BAM files of mapped reads were further used to annotate transcripts and to calculate the fragments per kilobase of transcript per million mapped reads using the Cufflinks, Cuffquant, Cuffnorm suite^34^.

### Differential gene expression analysis

Differential expression analysis was performed using R_3.3 and Bioconductor_3.4^35^. RNA-seq data were analysed using DESeq2_1.18.0^36^ package. Microarray data were analysed using limma_3.34.0 package^37^. A model formula of ~B + A was used for performing the analysis^36, 37^. The results of the effect of factor A can be adjusted by the condition of B. A stands for the major factor (IK6 expression or state of *IKZF1* alteration) in which we were interested and B stands for the condition (cell population or subtype of patients) we wanted to consider.

### GSEA

GSEA and leading-edge analysis were performed using GSEA_3.0 desktop application^38, 39^. GSEA was run against Msigdb_v6.0 gene set database in our analysis, and the number of permutations was set to 1000.

### Software used in plotting

Regular plots were generated in R_3.3 using corresponding packages. Three-dimensional scatter plots were generated using scatterplot3d_0.3–40 package, and bar plots, dot plots and two-dimensional scatter plots were generated using ggplot2_2.2.1 package. Heat maps were generated using pheatmap_1.0.8 package.

### Network analysis between the enriched gene sets

Network analysis and visualisation were conducted in Cytoscape_3.5.1^40^ desktop software in combination with EnrichmentMap_3.0.0^41^ plugin. An enrichment map network of GSEA results was generated using the gene sets with an enrichment *P*-value of <0.05 and FDR of <0.1, and an association coefficient threshold of ≤0.5. A node represents a gene set, and the size of node was mapped to the size (number of genes involved in the gene set) of corresponding gene set, the node colour saturation was mapped to the normalised enrichment score of the corresponding gene set, and the node shape was mapped to the group to which the corresponding gene set belonged. An edge represented the association between the vertices’ gene sets, and the width of the edge was mapped to the association coefficient. The intergroup association was defined as the sum of the edges.

### URLs

R https://www.R-project.org/.

Bioconductor http://www.bioconductor.org/.

DESeq2 https://bioconductor.org/packages/release/bioc/html/DESeq2.html.

limma https://bioconductor.org/packages/release/bioc/html/limma.html.

GSEA http://software.broadinstitute.org/gsea/.

scatterplot3d https://cloud.r-project.org/web/packages/scatterplot3d.

ggplot2 http://ggplot2.tidyverse.org/.

Pheatmap https://cloud.r-project.org/web/packages/pheatmap/.

Cytoscape http://www.cytoscape.org/.

EnrichmentMap http://www.baderlab.org/Software/EnrichmentMap.

GeneMANIA https://www.genemania.org/.

ENCODE https://www.encodeproject.org/.

### Accession code

RNA–seq data have been deposited at the National Center for Biotechnology Information under accession number GSE112124.

### Data availability

All data supporting the findings of this study are available from the corresponding author upon reasonable request.

## Electronic supplementary material

Supplementary

Supplementary figure 1

Supplementary figure 2

Supplementary figure 3

Supplementary table 1

Supplementary table 2
