# Supplementary material for: Leukaemic alterations of IKZF1 prime stemness and malignancy programs in human lymphocytes
Source: Cell Death Dis. 2018 May 9;9(5):526. doi: 10.1038/s41419-018-0600-3 (PMC5943605; doi:10.1038/s41419-018-0600-3)
Supplement: Supplementary file 1 — Supplementary [file 41419_2018_600_MOESM1_ESM.docx]

**Supplementary Figure Legends**

**sFig 1. Occurrence of *IKZF1* alterations and Gene Set Enrichment Analysis (GSEA) in the archived patient datasets. (a)** Occurrence of *IKZF1* alterations in subtypes of acute lymphoblastic leukaemia (ALL) in patients on the basis of data collected in the TARGET program and Pediatric Cancer Genome Project (PCGP). **(b)** t-Distributed Stochastic Neighbor Embedding analysis for the data of samples with *IKZF1* alterations in the GSE11877 dataset and mapped sample sources (peripheral blood and bone marrow). **(c)** GSEA analysis of the EGAD00001001016 dataset^16, 23^ of contrasting samples with/without *IKZF1* alteration. Note: haematopoietic stem cell (HSC) and leukemic stem cell (LSC) programs were upregulated in cells with *IKZF1* alteration.

**sFig 2. Functional assay of IK6 in human haematopoietic cells *in vivo*. (a)** The complete coding sequence of IK6. **(b)** cDNA of IK6 was cloned into the pHR-SIN-CSGW lentivirus vector^1^. **(c)** Western blot analysis of IK6 expression in CD34^+^ umbilical CB cells at 3 days after infection. **(d)** Engraftment of control vector (Ctrl) and IK6-transduced CB haematopoietic stem and progenitor cells (HSPCs) in the primary recipient mice detected by flow cytometry using anti-human CD45 and the results were statistically summarised. **(e)** Flow cytometric analysis of the GFP^+^ cells in engrafted cells (CD45) in the primary recipients. The percentage of GFP^+^ cells in total CD45^+^ cells was labelled. **(f)** Flow cytometric analysis of the engraftment contribution of GFP^+^ cells in primary mice and infection efficiency before transplantation (input). **(g)** Within the engrafted GFP^+^ cells, the repopulation of B cells (CD19^+^), T cells (CD3^+^) and myeloid cells (CD33^+^) was analysed by flow cytometry and the results were statistically summarised.

**sFig 3. Transcriptome profiling of the cell populations isolated from the first recipient (1^st^) mice. (a)** Consistency analysis of the RNA-seq data in the three samples of independent experiments within each group. **(b)** Unsupervised hierarchical clustering analysis of the Ctrl groups (G1, G2 and G3) compared with the groups flow sorted from umbilical CB cells [i.e. CB haematopoietic stem cell (HSC) and CB B cells]. Note that Ctrl G1 was close to CB HSCs, and Ctrl G2 and Ctrl G3 were close to CB B cells. **(c and d)** Gene set enrichment analysis of the whole transcriptome among Ctrl G1, G2 and G3. Note that from G1 to G3, the stem cell programs were downregulated and B cell programs were upregulated. **(e)** Top 100 genes proposed as having possible physical interaction with *IKZF1* were retrieved from GeneMANIA database. Unsupervised hierarchical clustering analysis of these genes in our RNA-seq dataset revealed minimal differences between IK6 samples and Ctrl samples. **(f)** Genes that are potentially regulated by *IKZF1* were retrieved from ENCODE_TF_ChIP-seq database and analysed in our dataset. In total, 239 of these genes showed significant differential expression between the IK6 and Ctrl groups.
