# Supplementary figures and images for: Leukaemic alterations of IKZF1 prime stemness and malignancy programs in human lymphocytes
Source: Cell Death Dis. 2018 May 9;9(5):526. doi: 10.1038/s41419-018-0600-3 (PMC5943605; doi:10.1038/s41419-018-0600-3)

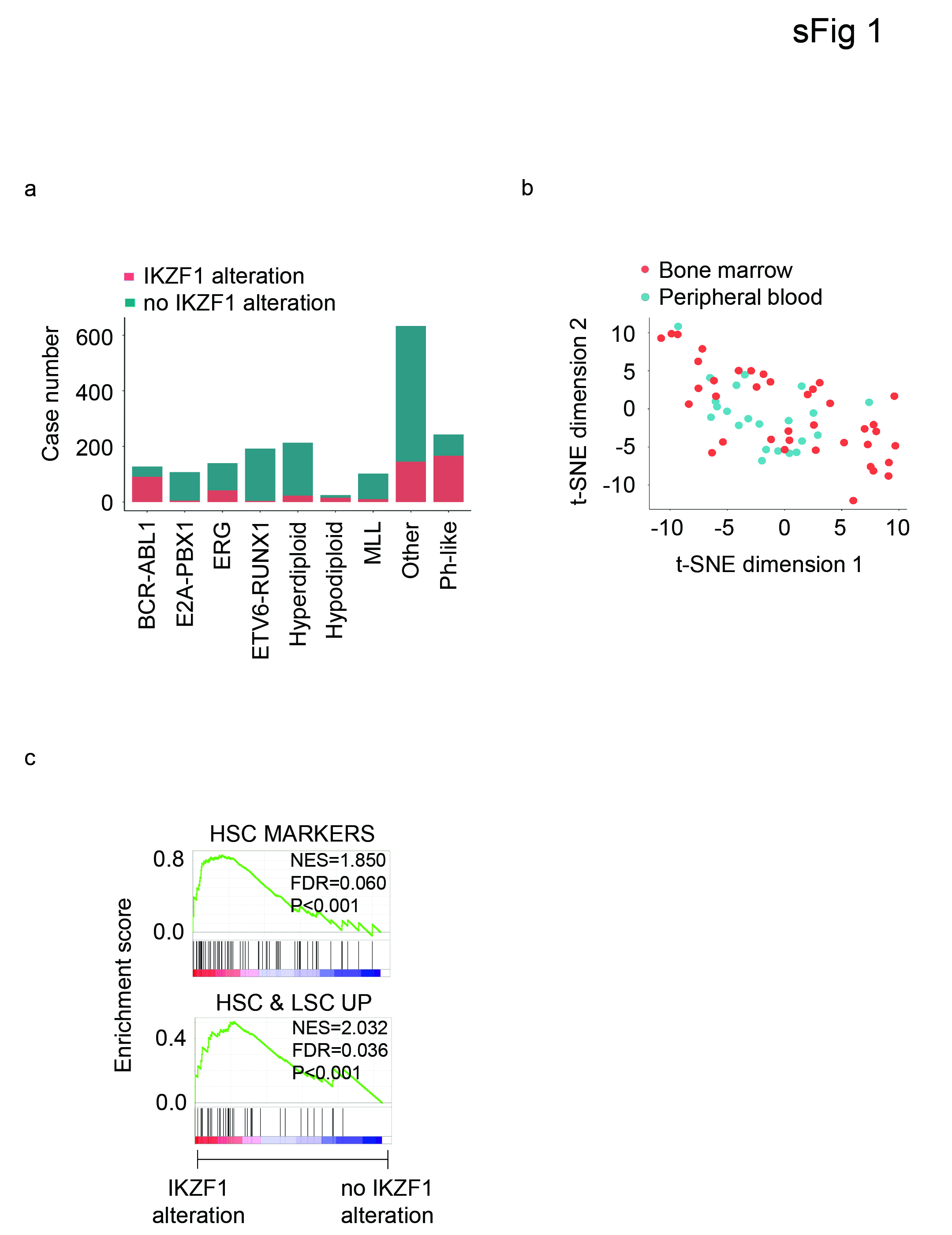

Supplement: Supplementary file 2 — Supplementary figure 1 [file 41419_2018_600_MOESM2_ESM.tif]

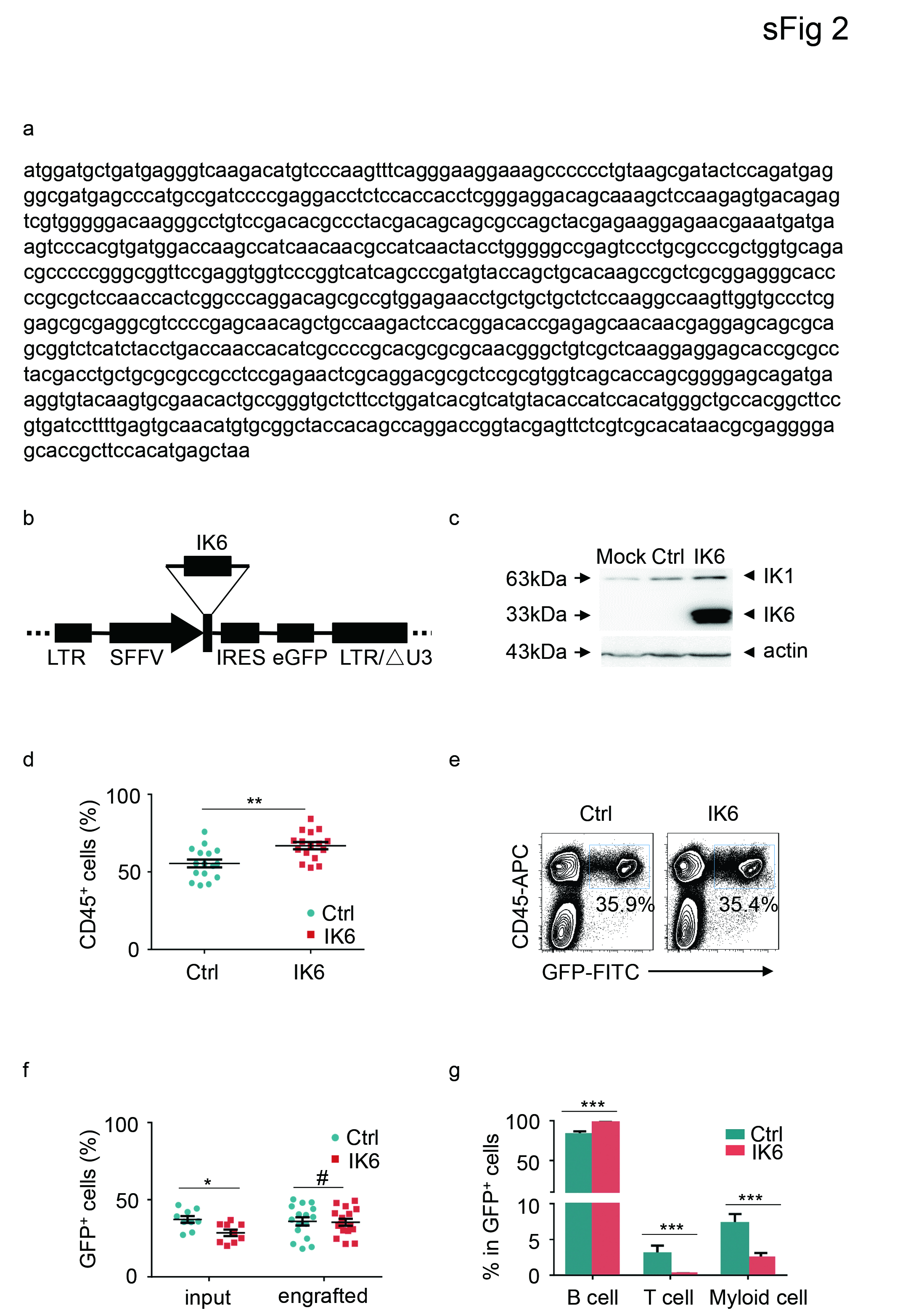

Supplement: Supplementary file 3 — Supplementary figure 2 [file 41419_2018_600_MOESM3_ESM.tif]

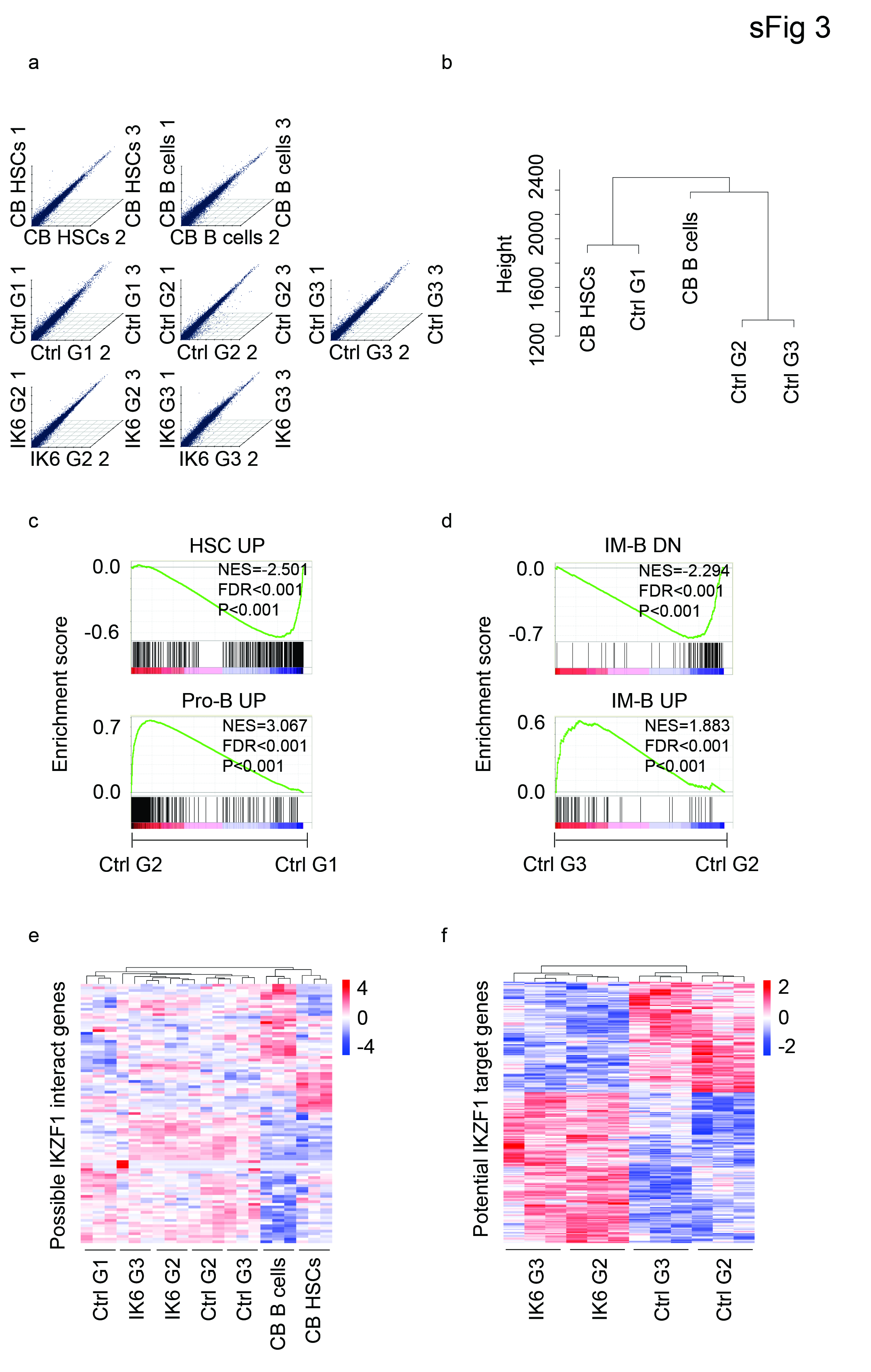

Supplement: Supplementary file 4 — Supplementary figure 3 [file 41419_2018_600_MOESM4_ESM.tif]
